# UPLC-ESI-TQD-MS/MS Identification and Antioxidant, Anti-Inflammatory, Anti-Diabetic, Anti-Obesity and Anticancer Properties of Polyphenolic Compounds of Hawthorn Seeds

**DOI:** 10.1007/s11130-024-01197-4

**Published:** 2024-05-30

**Authors:** Natalia Żurek, Michał Świeca, Ireneusz Kapusta

**Affiliations:** 1 Department of Food Technology and Human Nutrition, College of Natural Sciences, University of Rzeszow, 4 Zelwerowicza St., Rzeszow, 35-601 Poland; 2 Department of Food Chemistry and Biochemistry, University of Life Sciences in Lublin, 8 Skromna St., Lublin, 20-704 Poland

**Keywords:** Seeds, *Crataegus* L., Hawthorn, Pro-health activity, UPLC, Polyphenol content

## Abstract

Hawthorn seeds are a by-product of fruit processing and due to the scale of processing of this raw material, they can be an important source of bioactive compounds. This work is the first report on the phenolic composition of hawthorn seeds and their antioxidant, anti-inflammatory, antidiabetic, antiobesity and anticancer activities. In the isolated phenolic fraction of six seed species, 23 phenolic compounds were identified using the UPLC-ESI-TQD-MS/MS method, the key ones of which included the B-type procyanidin dimer. The seeds of the tested species showed high antioxidant activity (mainly by scavenging O_2_^•−^ and OH^•^ radicals), anti-inflammatory (mainly through LOX inhibition), anti-diabetic, anti-obesity and anti-cancer, with the highest activity against colon cancer cells (Dld-1 line), showing no activity against healthy colon epithelial cells (CCD841CoN). This activity was significantly dependent on the analyzed hawthorn species and, according to PCA analysis, on the content of flavan-3-ols. These discoveries provided the theoretical basis for the possibility of industrial use of hawthorn seeds.

**Graphical Abstract:**

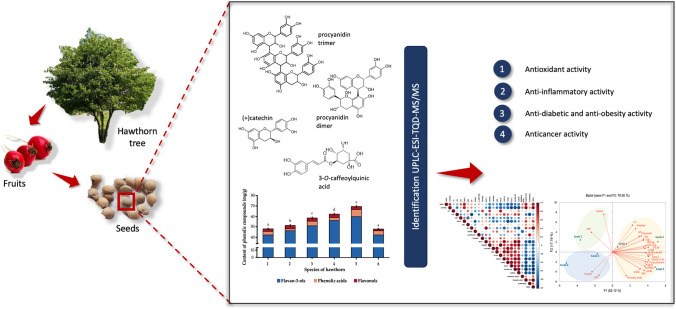

**Supplementary Information:**

The online version contains supplementary material available at 10.1007/s11130-024-01197-4.

## Introduction

Hawthorn (*Crataegus* L.) belongs to the rose family (*Rosaceae*) and the apple subfamily (*Maliodeae*). The estimated number of hawthorn species in the world ranges from 20 to 185. This plant is widely distributed in North America, Europe, Central and East Asia [1]. Currently, the use of hawthorn as a natural health product has been recognized by the Pharmacopoeia. The medicinal raw material is the inflorescence (*Crataegi folium cum florae*) and fruit (*Crataegi fructus*) collected at full ripeness and dried [1, 2]. Hawthorn is considered one of the most valuable plant raw materials used in the treatment of cardiovascular diseases, and the beneficial health-promoting effects of this plant have been attributed to phenolic compounds [3–5]. The fruits are dominated by oligomeric procyanidins and their glycosides, while the leaves and flowers are dominated by phenolic acids and flavonols.

However, compared to the morphological parts of the hawthorn bush discussed above, incomparably less research has been carried out on the chemical composition and possible health-promoting activity of hawthorn seeds. In Asia alone, the annual production of hawthorn exceeds 2.1 million tons [6]. A by-product of medicinal and food processing of fruit are seeds, which, due to the scale of processing of this raw material, become available in huge quantities. Therefore, their full phytochemical characterization seems necessary to fully exploit their possible health-promoting potential.

Therefore, the aim of the study was to investigate the content of bioactive ingredients in the isolated phenolic fraction of the seeds of six species of hawthorn and their impact on the antioxidant (scavenging of ABTS, O_2_^•^^−^, OH^•^ radicals, cupric reducing antioxidant capacity and chelation of iron ions), anti-inflammatory (LOX, COX-2, XO, trypsin inhibition), anti-diabetic (α-amylase), anti-obesity (lipase), anticancer against ten human cancer cell lines (Mcf-7, U87mg, U251mg, AGS, Ht-29, Caco-2, Ls180, Dld-1, Sk-mel-28, Jurakt) and cytotoxic to a healthy colonic epithelial cell line (CCD841CoN). Moreover, for better understanding of the data, Pearson correlation and PCA test were performed. The obtained results constitute the first comprehensive analysis of the polyphenol profile and health-promoting properties of hawthorn seeds, so they can be a good reference for the use of the bioactive substances they contain in the medical, cosmetic and food industries.

## Materials and Methods

The material and methods section are presented as Supplementary Material.

## Results and Discussion

### Phenolic Profile

**Fig. 1:**
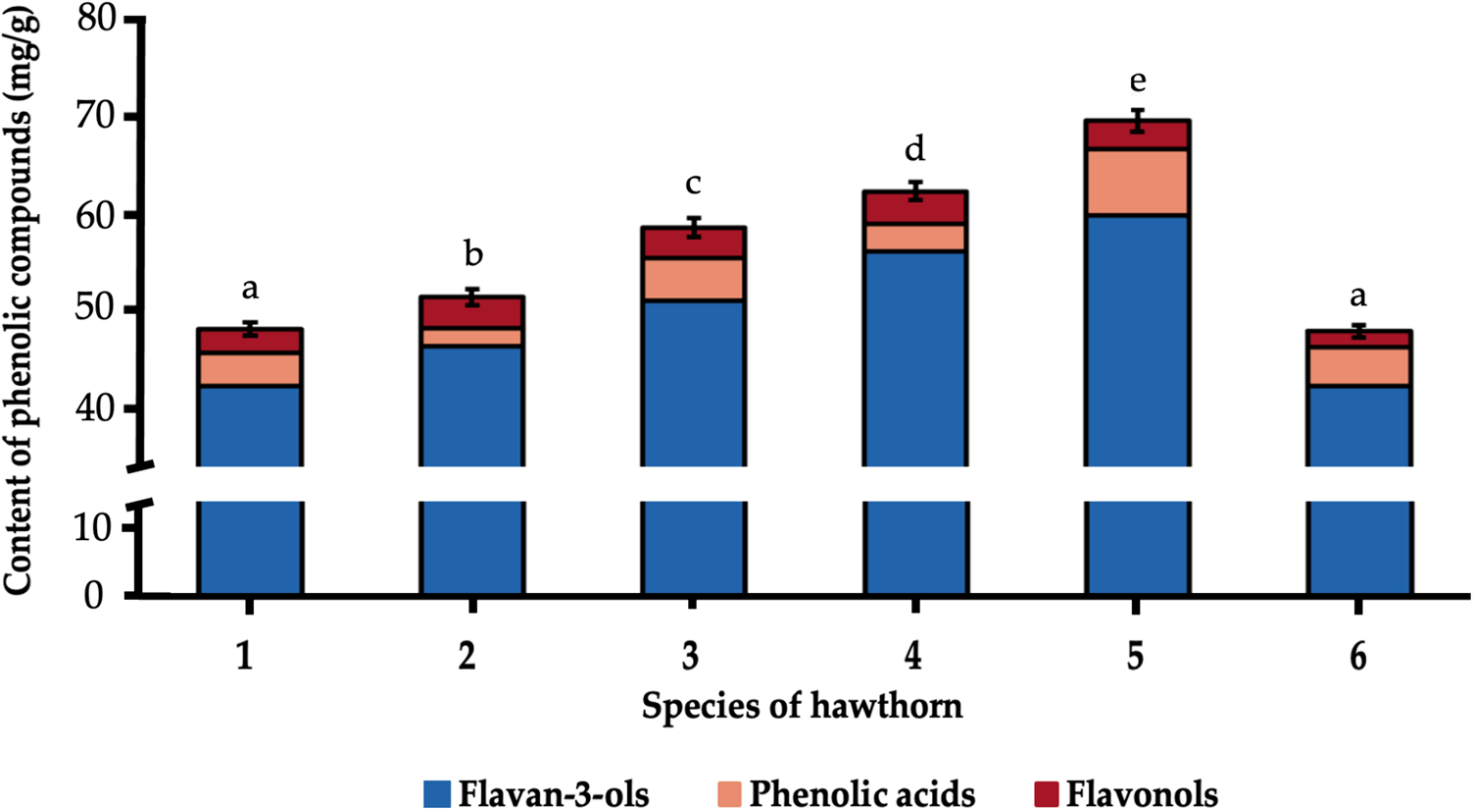
Content of three groups of phenolic compounds (flavan-3-ols, phenolic acids, flavonols) identified in the seeds of six hawthorn species. Hawthorn species: 1, *C. monogyna*; 2, *C. rhipidophylla*; 3, *C. x subsphaericea*; 4, *C. laevigata x rhipidophylla x monogyna*; 5, *C. macrocarpa*; 6, *C. laevigata*

As mentioned, the proven clinical medicinal activity of hawthorn flowers and fruits has been attributed to the content of phenolic compounds. Therefore, the first step in the analysis of hawthorn seeds was to assess the phenolic profile. Depending on the species, the content of phenolic compounds ranged from 46.8 (*C. laevigata*) to 71.3 (*C. macrocarpa*) mg/g (Fig. 1, Table 1). 23 compounds were identified in the phenol profile, of which 16 belonged to the flavan-3-ols group, 4 to flavonols and 3 compounds to the phenolic acids group. The quantitative profile of these groups of compounds was as follows: 84.6 – 89.0% (flavan-3-ols) > 4.1 – 10.8% (phenolic acids) > 3.9 – 7.2% (flavonols). The content of individual phenolic compounds in the seeds of 6 hawthorn species is given in Table S2. The most numerous group of phenolic compounds in hawthorn seeds were flavan-3-ols, such as polymeric procyanidins. Their amount ranged from 40.4 (*C. monogyna*; *C. laevigata*) to 60.3 (*C. macrocarpa*) mg/g. Among the identified flavan-3-ols, the highest concentrations were: procyanidin dimer type-B (27.1—37.1%) > procyanidin trimer type-C (26.1—29.2%) > (+)catechin (19.2—28.1%). Another group of phenolic compounds identified in hawthorn seeds are phenolic acids. Their amount ranged from 2.1 (*C. rhipidophylla*) to 7.7 (*C. macrocarpa*) mg/g. Among the identified phenolic acids, the highest concentrations were: 3-*O*-caffeoylquinic acid, 5-*O*-caffeoylquinic acid and coumarylquinic acid, in the following proportions: 19.6 – 68.0% > 16.4 – 64.4% > 15.6 – 45.2%. The third group of compounds found in hawthorn seeds were flavonols at concentrations ranging from 1.8 (*C. laevigata*) to 3.8 (*C. laevigata x rhipidophylla x monogyna*) mg/g. The compound from this group that dominated in five hawthorn species was kaempferol-*O*-galloyl-pentoside isomer II (30.3 – 38.0%). Next, there were: kaempferol-*O*-galloyl-pentoside isomer I (27.8—31.4%) and quercetin-*O*-acetyl-hexoside (20.8—28.1%).

**Table 1:** Individual phenolic compounds identified by UPLC-ESI-TQD-MS/MS in seeds hawthorn

	Compound	Rt	λ_max_	Molecular Formula	Exact mass	[M-H]^−^ m/z	
		min	nm			MS	MS/MS
1	5*-O*-caffeoylquinic acid^*^	2.35	324	C_16_H_18_O_9_	354.3093	353	191
2	Procyanidin dimer type-A	2.45	279	C_30_H_24_O_12_	576.5055	573	447, 288
3	Coumarylquinic acid	2.86	310	C_16_H_18_O_8_	338.3099	337	191
4	3-*O*-caffeoylquinic acid^*^	2.96	324	C_16_H_18_O_9_	354.3093	353	191
5	Procyanidin trimer	3.10	278	C_45_H_38_O_18_	866.7741	865	577
6	Procyanidin dimer type-B	3.16	278	C_30_H_26_O_12_	578.5213	577	425, 289
7	Procyanidin dimer type-B	3.23	278	C_30_H_26_O_12_	578.5213	577	425, 289
8	(+)Catechin^*^	3.48	278	C_15_H_14_O_6_	290.2686	289	-
9	Proanthocyanidin dimer	3.57	278	C_30_H_24_O_12_	578.5213	579	449, 287
10	Procyanidin trimer	3.62	278	C_45_H_38_O_18_	866.7741	865	577
11	Procyanidin dimer type-B	3.74	278	C_30_H_26_O_12_	578.5213	577	425, 289
12	Proanthocyanidin pentamer I	3.79	279	C_75_H_62_O_30_	1443.2796	1441	865, 577
13	Procyanidin trimer	3.87	278	C_45_H_38_O_18_	866.7741	865	577
14	Proanthocyanidin pentamer II	4.34	279	C_75_H_62_O_30_	1443.2796	721	865, 577
15	Procyanidin trimer	4.46	278	C_45_H_38_O_18_	866.7741	865	577
16	Procyanidin dimer type-B	4.55	278	C_30_H_26_O_12_	578.5213	577	425, 289
17	Procyanthocyanidin trimer	4.67	278	C_45_H_38_O_18_	866.7741	863	575
18	(Epi)afzelechin-di-hexoside	4.92	276	C_27_H_34_O_15_	598.5501	597	435, 273
19	Unspecified (Epi)afzelechin derivative	4.98	276	-	-	551	405, 273
20	Kaempferol-*O*-galloyl-pentoside isomer I	5.61	285, 337	C_27_H_22_O_14_	570.4562	569	417
21	Kaempferol-*O*-galloyl-pentosideisomer II	5.68	285, 337	C_27_H_22_O_14_	570.4562	569	417
22	Quercetin *O*-acetyl-hexoside	7.41	281, 345	C_23_H_22_O_13_	506.4139	505	463, 301
23	Unspecified	7.76	278, 338	-	-	779	617, 581

*-compared with authentic standards

So far, the phenolic profile of hawthorn seeds has been analyzed in only two studies. Salmanian et al. [7] and Niu et al. [8] identified three compounds in *C. elbursensis* seeds, such as caffeic acid, chlorogenic acid and gallic acid, with a total concentration 41.2 times lower than in this study. These differences may be due to the extraction method used in this work. As a result of extraction into the solid phase, the obtained preparation was cleaned of ballast substances, which resulted in an increase in the phenolic fraction. In our studies, phenolic acids accounted for only 4.1 – 10.8% of the total identified phenols, and their content between species differed statistically significantly (*p* < 0.05). Nevertheless, this group of compounds dominates the phenolic composition of hawthorn flowers and leaves, accounting for 53 and 41% of all phenols, respectively [9]. Żurek et al. concluded that the dominant compound in these parts of the plant is 3-*O*-caffeoylquinic acid [10]. In turn, the dominant compounds in hawthorn fruits are flavan-3-ols, which constitute nearly 55% of their phenolic composition, and the largest amounts include: procyanidin dimer, procyanidin trimer and (+)catechin [9]. Their total amount in hawthorn fruits is 1.3 times higher than in the tested seeds. Overall, the procyanidin fraction of hawthorn fruit is of great interest as important ingredients in nutrition with high biological activity, including antioxidant, antibacterial, antiviral, anticancer, anti-inflammatory, neuro- and cardioprotective activities [11]. Han et al. [12] demonstrated a beneficial effect of procyanidin extract from hawthorn fruit on the intestinal microflora and the production of short-chain fatty acids in the colon. It is therefore worth noting that hawthorn seeds, a by-product of hawthorn fruit processing, are an important source of procyanidins, which, once isolated, can be used to develop preparations with targeted health-promoting effects.

## Antioxidant Activity

Values for the ABTS and CUPRAC methods ranged from 5.8 to 8.2 mmol TE/g and from 9.6 to 12.9 mmol TE/g, respectively. O_2_^•−^ and OH^•^ radical scavenging activities ranged from 65.4 to 135.0 μg/mL and from 71.4 to 175.1 μg/mL, respectively (Table 2). In turn, for the ChP method the values ranged from 70.0 to 452.3 μg/mL. The antioxidant activity of the analyzed species varied and showed significant differences (*p* < 0.05). The highest antioxidant activity was found for the seeds of *C. macrocarpa* (ABTS, CURPAC method), *C. laevigata x rhipidophylla x monogyna* (O_2_^•−^ and OH^•^) and *C. monogyna* (ChP).

**Table 2:** Antioxidant, anti-inflammatory, anti-obesity, anti-diabetes, anticancer activity of seeds of six hawthorn species

	Species of hawthorn					
	*C. monogyna*	*C. rhipidophylla*	*C. x subsphaericea*	*C. laevigata x rhipidophylla x monogyna*	*C. macrocarpa*	*C. laevigata*
Antioxidant activity						
ABTS [mmol TE/g]	5.8 ± 0.3^a^	5.8 ± 0.4^a^	5.9 ± 0.1^a^	7.92 ± 1.1^b^	8.2 ± 1.0^b^	6.0 ± 0.3^a^
CUPRAC [mmol TE/g]	9.9 ± 0.1^ab^	10.4 ± 0.1^c^	10.3 ± 0.1^bc^	11.7 ± 0.5^d^	12.9 ± 0.2^e^	9.6 ± 0.1^a^
O_2_^•−^ [IC_50_, µg/mL]	68.2 ± 1.2^a^	161.0 ± 2.5^d^	103.1 ± 3.5^b^	65.4 ± 0.7^a^	104.1 ± 1.9^b^	135.0 ± 1.1^c^
OH^•^ [IC_50_, µg/mL]	130.7 ± 0.8^d^	97.9 ± 2.5^b^	129.4 ± 1.6^d^	71.4 ± 0.8^a^	104.6 ± 1.7^c^	175.1 ± 1.3^e^
ChP [IC_50_, µg/mL]	70.0 ± 0.4^a^	89.5 ± 1.8^b^	132.4 ± 3.2^c^	452.3 ± 1.5^e^	149.0 ± 2.0^d^	148.3 ± 2.5^d^
Pro-health activity						
LOX inhibition[MIU/g]	346.2 ± 10.6^b^	374.6 ± 7.1^b^	373.9 ± 13.9^b^	285.5 ± 25.2^a^	375.7 ± 14.8^b^	362.3 ± 11.1^b^
COX-2 inhibition [MIU/g]	2.1 ± 0.6^a^	4.1 ± 0.6^b^	2.6 ± 0.6^a^	3.9 ± 0.3^b^	6.1 ± 0.6^c^	6.0 ± 0.5^c^
XO inhibition [IU/g]	141.6 ± 1.1^b^	147.1 ± 1.9^ cd^	146.6 ± 1.6^c^	149.6 ± 1.1^d^	135.7 ± 1.1^a^	146.1 ± 1.9^c^
Lipase inhibition [kIU/g]	16.9 ± 1.8^e^	12.0 ± 1.9^bc^	14.1 ± 1.8^ cd^	14.3 ± 0.6^d^	8.7 ± 1.2^a^	10.5 ± 1.3^b^
α-amylase inhibition [kIU/g]	7.6 ± 1.0^b^	6.5 ± 0.3^b^	7.4 ± 1.0^b^	7.6 ± 0.7^b^	6.9 ± 1.2^b^	3.8 ± 0.6^a^
Trypsin inhibition [kIU/g]	8.9 ± 1.1^bc^	11.1 ± 0.8^d^	7.8 ± 0.6^a^	8.2 ± 0.4^ab^	9.3 ± 1.0^bc^	10.8 ± 0.1^ cd^
Anticancer activity						
Mcf-7 [IC_50_, µg/mL]	151.4 ± 5.6^b^	193.6 ± 5.0^c^	215.8 ± 2.2^e^	207.3 ± 2.8^d^	204.1 ± 3.1^d^	137.1 ± 3.4^a^
U87mg [IC_50_, µg/mL]	190.6 ± 3.0^c^	206.6 ± 2.2^d^	183.8 ± 1.0^b^	169.2 ± 1.7^a^	171.5 ± 0.9^a^	203.0 ± 4.6^d^
U251mg [IC_50_, µg/mL]	698.8 ± 4.2^f^	472.6 ± 3.4^e^	356.3 ± 6.6^c^	267.9 ± 9.0^a^	332.1 ± 5.1^b^	378.4 ± 11.9^d^
AGS [IC_50_, µg/mL]	267.1 ± 8.9^e^	205.1 ± 5.1^c^	200.0 ± 3.1^bc^	189.8 ± 4.1^ab^	186.8 ± 5.0^a^	223.8 ± 6.8^d^
Ht-29 [IC_50_, µg/mL]	157.1 ± 5.5^b^	212.4 ± 6.6^d^	154.8 ± 3.5^b^	109.0 ± 3.3^a^	105.9 ± 4.2^a^	201.9 ± 4.0^c^
Caco-2 [IC_50_, µg/mL]	176.8 ± 6.6^d^	133.6 ± 5.2^c^	115.7 ± 2.6^b^	104.3 ± 2.7^a^	111.1 ± 3.2^ab^	171.7 ± 1.6^d^
Ls180 [IC_50_, µg/mL]	184.2 ± 2.3^c^	159.5 ± 2.0^b^	159.5 ± 3.0^b^	134.9 ± 6.0^a^	134.3 ± 2.9^a^	162.6 ± 1.3^b^
Dld-1 [IC_50_, µg/mL]	200.9 ± 3.8^e^	192.0 ± 4.9^d^	159.7 ± 5.8^c^	121.7 ± 2.4^b^	94.6 ± 5.8^a^	166.1 ± 4.1^c^
Sk-mel-28 [IC_50_, µg/mL]	714.6 ± 3.8^e^	619.3 ± 1.8^d^	579.5 ± 4.0^c^	525.9 ± 3.8^b^	459.3 ± 4.4^a^	735.3 ± 3.2^f^
Jurkat [IC_50_, µg/mL]	337.9 ± 2.1^c^	331.2 ± 4.5^c^	296.4 ± 7.4^b^	278.2 ± 8.1^a^	280.2 ± 8.5^a^	361.3 ± 8.0^d^
CCD841CoN [IC_50_, µg/mL]	> 750	> 750	> 750	> 750	> 750	> 750

Results are expressed as mean and SD. Significant differences between species were assessed by Duncan’s test (*p* < 0.05)

**Fig. 2:**
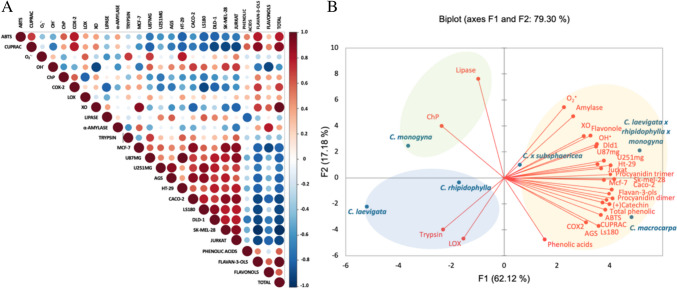
**A** Pearson correlation showing the relationships between the studied variables. **B** Principal component analysis (PCA) of data on the content of phenolic compounds and health-promoting activity of seeds of six species of hawthorn. Hawthorn species: 1, *C. monogyna*; 2, *C. rhipidophylla*; 3, *C. x subsphaericea*; 4, *C. laevigata x rhipidophylla x monogyna*; 5, *C. macrocarpa*; 6, *C. laevigata*

The antioxidant activity of hawthorn seeds has not been assessed so far by any of the above methods. The available literature only contains data expressing the antioxidant activity of three parts of hawthorn fruit using the DPPH and FRAP test, in the order peel > seed > pulp [7, 13]. In turn, compared to other morphological parts of hawthorn, in relation to the present results, the antioxidant activity of the fruit was lower by 28.3 times (for the ABTS test) [14], 4.1 times (ChP) [15], 3.0 times (O_2_^•−^) and 1.2 times higher (OH^•^) [16]. These significant differences in antioxidant activity can be attributed to the method of obtaining preparations for analysis. The cited works assessed mainly the so-called crude extracts, most often ethanol. However, in this study, the preparations were obtained as a result of SPE extraction, which allowed the removal of ballast substances, which resulted in an increased content of phenols and antioxidant activity. The relationship between antioxidant activity and the content of phenolic compounds was also confirmed by Pearson’s correlation. A significant correlation was observed between the total content of phenols and flavan-3-ols and the ABTS test (flavan-3-ols r > 0.794; total phenols r > 0.781) and the CUPRAC test (flavan-3-ols r > 0.930; total phenols r > 0.938) and between flavonols and OH^•^ scavenging (r > -0.889) (Fig. 2A). Similar observations apply to hawthorn fruits, for which a strong correlation with procyanidins was demonstrated, indicating that procyanidin B2 and (-)epicatechin are the most effective antioxidant compounds in hawthorn [17, 18].

### Anti-Diabetic and Anti-Obesity Activity

The lipase inhibition activity ranged from 8.7 – 16.9 kIU/g, α-amylase activity ranged from 3.8 – 7.6 kIU/g, and trypsin activity ranged from 7.8 – 11.1 kIU/g (Table 2). The highest activity was demonstrated for seeds of the species *C. monogyna* (lipase, α-amylase), *C. laevigata x rhipidophylla x monogyna* (α-amylase) and *C. rhipidophylla* (trypsin). In the case of lipase, there were no significant differences (*p* < 0.05) between the five species.

So far, it has only been reported that polysaccharides isolated from *C. azarolus* seeds have a threefold lower ability to inhibit α-amylase (IC_50_, 3.01 mg/mL) than pulp polysaccharides [19]. There are no reports in the literature about the lipase and trypsin-inhibiting effects of hawthorn extracts. In this study, the activity of α-amylase inhibition was shown to be correlated with polyphenolic compounds, more precisely with flavonols (r > 0.805). In studies conducted on rats, the activity in the treatment of hyperglycemia was also attributed to hawthorn fruit flavonoids, mainly quercetin and hyperoside [20, 21]. The authors showed that extracts rich in these ingredients lower blood glucose levels, increase insulin release and inhibit the increase in postprandial glucose levels. However, weak correlations were noted for lipase and trypsin (r < 0.217). Therefore, the lack of relationship between the inhibition of the activity of these enzymes and the content of polyphenols may indicate that this activity may be influenced by other groups of compounds. Previously, Wojdyło et al. showed that terpenoids and alkaloids are strong lipase and trypsin inhibitors [22].

### Anti-Inflammatory Activity

The highest LOX and COX-2 inhibition activity was demonstrated for the species *C. macrocarpa*. The obtained values ranged from 285.5 to 375.7 MIU/g and 2.1 – 6.1 MIU/g, respectively (Table 2). In the case of XO, the inhibitory effect was much lower compared to COX-2 and LOX, and ranged from 135.7—149.6 IU/g. The highest activity was demonstrated for the species *C. laevigata x rhipidophylla x monogyna*. Generally, no significant differences between species were found in the LOX test. The demonstrated anti-inflammatory activity (for the COX-2 and XO tests) can be attributed to polyphenolic compounds, mainly the content of flavan-3-ols (Pearson correlation COX-2 vs. flavan-3-ols, r > 0.730; XO vs. flavan- 3-ols, r > 0.658) and total phenols (COX-2 vs. total phenols, r > 0.686; XO vs. total phenols, r > 0.968). The relationship between anti-inflammatory activity and phenolic content has been reported previously. Cui et al. [23] and Wyspiańska et al. [24] examined the anti-inflammatory effects of hawthorn fruit and bark. They determined that the anti-inflammatory activity of these raw materials is correlated with the content of procyanidins, in particular epicatechin and procyanidin B. Also, Elango and Devaraj [25] for procyanidins extracted from hawthorn fruit showed the ability to alleviate the pro-inflammatory immune response in rats by significantly reducing the level of pro-inflammatory mediators (IL-1β, IL-6 i TNF-α). In general, the anti-inflammatory activity of procyanidins has been confirmed in numerous studies conducted on both animal and human cellular models of inflammation [26].

### Cell Viability

In terms of the cytotoxic properties of hawthorn seeds towards the analyzed cancer lines, the assessed activity can be arranged in the following order: Dld-1 > Caco-2 > Ht-29 > Ls180 > U87mg > AGS > Mcf-7 > Jurakt > U251mg > Sk-mel-28 (Table 2). The highest anticancer activity was demonstrated against colorectal cancer cells. The obtained IC_50_ values ranged from 94.6 to 200.9 (Dld-1) and 105.9 – 212.4 (Ht-29) μg/mL. With respect to these cell lines, the highest activity was demonstrated by seeds of the species *C. laevigata x rhipidophylla x monogyna* and *C. macrocarpa*, and the lowest by *C. monogyna* and *C. rhipidophylla*. In turn, the lowest activity was found against malignant cancer cell lines, such as Sk-mel-28 and U251mg, which are characterized by high therapeutic resistance. This is the first report on the anticancer activity of hawthorn seeds.

Other authors examining hawthorn fruits also reported the highest activity against colorectal cancer cells. It has been proven that hawthorn polysaccharides inhibit the proliferation of colon cancer cells (HCT116) by arresting the cell cycle in the G2/M and S phase and inducing apoptosis as a result of activation of P38 kinase [27]. In our previous work, we showed that among the fruits, leaves and flowers of hawthorn, the fruits have the strongest anticancer activity [9]. Their activity against glioblastoma cells (U87mg) depended on the concentration of flavan-3-ols and caused cell apoptosis by cutting PARP1, inhibiting the activity of FAK and Akt kinases, indicating the suppression of the proliferative and invasive potential. Also in this study, the anticancer activity was strongly dependent on the content of flavan-3-ols. Between this group of compounds and all analyzed cancer cell lines, the Pearson correlation coefficient was r > -0.695. It is worth emphasizing that hawthorn seeds did not show any cytotoxicity towards normal colonic epithelial cells (> 750 µg/mL), which indicates the safety of use of the obtained preparations.

#### PCA

The PCA plot presented 79.30% of the total variance in the data, with PC1 and PC2 explaining 62.12% and 17.18% of the total variance, respectively (Fig. 2B). The first group contained seeds of the species *C. laevigata x rhipidophylla x monogyna*, *C. macrocarpa* and *C. subsphaericea*, which were characterized by a high content of flavonols, phenolic acids, flavan-3-ols (procyanidin dimer type-B, (+)catechin, procyanidin trimer), as well as high antioxidant activity, expressed by the ABTS, CUPRAC, O_2_^•−^ and OH^•^ method, XO and COX-2 inhibition, anti-diabetic and anticancer activity. The second group included the species *C. monogyna*, which was characterized by high anti-obesity and metal ion chelating activity. The third group consisted of seeds of *C. rhipidophylla* and *C. laevigata* species, which were characterized by anti-inflammatory activity (LOX and trypsin inhibition). It can be assumed that the health-promoting activity of seeds belonging to scaled groups 2 and 3 depended on ingredients other than phenolic compounds. Overall, the PCA analysis confirmed our previous conclusions, showing that the hawthorn species had a significant impact on the content of phenolic compounds and health-promoting activity.

## Conclusion

This study provided missing data on the phenolic composition of hawthorn seeds and their broad health-promoting activities *in vitro*. The evaluated hawthorn seeds were particularly rich in flavan-3-ols, which constituted 84.6 – 89.0% of the total quantitative phenolic composition, where the dominant compounds were procyanidin dimer type-B and procyanidin trimer type-C. The highest concentration of these compounds was recorded for the species *C. macrocarpa*. High antioxidant activity was observed for the tested seeds through various mechanisms of action, anti-diabetic, anti-obesity and anti-inflammatory activity, including the highest activity towards LOX. Cytotoxic activity towards cancer cells, in particular colon cells, was also found, with no toxicity towards healthy colon epithelial cells. The highest values of the mentioned health-promoting properties were found in seeds of the species *C. macrocarpa* and *C. laevigata x rhipidophylla x monogyna*, whose high activity correlated with the content of flavan-3-ols.

Due to current consumer demands, it is necessary to analyze and promote unconventional raw materials and their ingredients with high potential for industrial use. The obtained results should particularly arouse the interest of the medical, cosmetic and food industries in the use of bioactive ingredients and health-promoting properties of hawthorn seeds in the development of attractive products with potential health benefits.

## Supplementary Information

Below is the link to the electronic supplementary material.

Supplementary file1 (DOCX 434 kb)

## Data Availability

No datasets were generated or analysed during the current study.
