# Supplementary material for: UPLC-ESI-TQD-MS/MS Identification and Antioxidant, Anti-Inflammatory, Anti-Diabetic, Anti-Obesity and Anticancer Properties of Polyphenolic Compounds of Hawthorn Seeds
Source: Plant Foods Hum Nutr. 2024 May 30;79(3):594–600. doi: 10.1007/s11130-024-01197-4 (PMC11410894; doi:10.1007/s11130-024-01197-4)
Supplement: Supplementary file 1 — Supplementary file1 (DOCX 434 kb) [file 11130_2024_1197_MOESM1_ESM.docx]

*Supplementary meterials 1*

**UPLC-ESI-TQD-MS/MS identification and antioxidant, anti-inflammatory, anti-diabetic, anti-obesity and anticancer properties of polyphenolic compounds of hawthorn seeds**

Natalia Żurek^1*^, Michał Świeca^2^, Ireneusz Kapusta^1^

1. Department of Food Technology and Human Nutrition, College of Natural Sciences, University of Rzeszow, 4 Zelwerowicza St., 35-601 Rzeszow, Poland
2. Department of Food Chemistry and Biochemistry, University of Life Sciences in Lublin, 8 Skromna St., 20-704 Lublin, Poland

***** Correspondence: nzurek@ur.edu.pl, Tel. +48-17-785-5236,

**Graphical abstract**


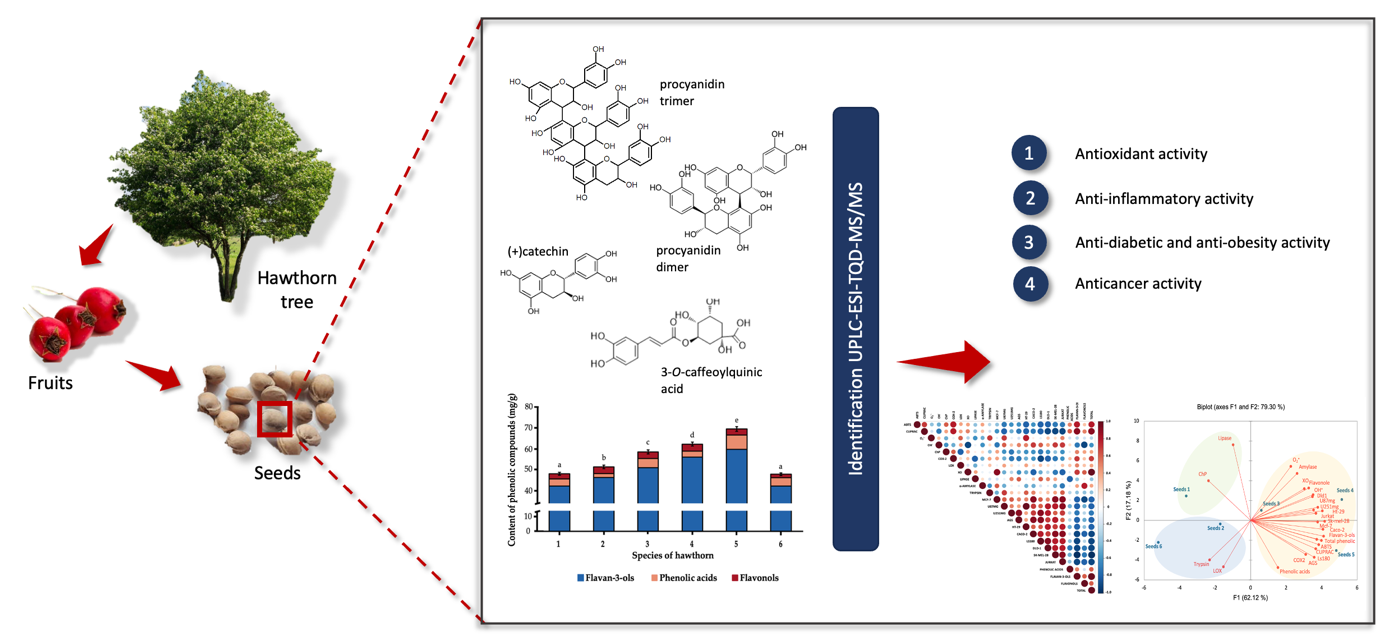


***Materials and Methods***

#### **Materials and Reagents**

Gallic acid (≥98%), LiChroprep RP-18 (40–63 µm), neocuproine (≥98%), ferrozine (≥97%), NBT (nitrotetrazolium blue chloride), PMS (phenazine methosulfate, ≥90%), NADH (*β*-Nicotinamide adenine dinucleotide, reduced disodium salt hydrate, ≥97%), 2-Deoxy-d-ribose, EDTA (ethylenediaminetetraacetic acid disodium salt dihydrate), Dulbecco’s Modified Eagle Medium, McCoy’s 5A Medium, fetal bovine serum, antibiotics (100 U/mL penicillin, 100 U/mL streptomycin), 0.25% trypsin–EDTA (1×), Dulbecco’s Phosphate Buffered Saline, and Mueller–Hinton Broth were purchased from Sigma-Aldrich (Darmstadt, Germany). The AQueous CellTiter 96 Non-Radioactive Cell Proliferation Test was purchased from Promega (Madison, WI, USA). Reference standard compounds for UPLC analyses were obtained from Extrasynthese (Lyon, France) and Sigma-Aldrich (Darmstadt, Germany). All other chemicals were purchased from Chempur (Piekary Śląskie, Poland).

***Plant Materials***

Seeds of six hawthorn species - *C. monogyna*, *C. rhipidophylla*, *C. x subsphaericea*, *C. laevigata x rhipidophylla x monogyna*, *C. macrocarpa* and *C. laevigata* - were obtained from the fruit of trees located in Błażowa (Poland). After fruit harvesting, the seeds were separated, freeze-dried (ALPHA 1-2 LD plus Martin Christ Gefriertrocknungsanlagen GmbH, Germany), and ground using a coffee grinder.

***Preparation of Extract***

Hawthorn seed extracts were prepared according to our previous methods [1, 2]. Briefly, the crushed material was subjected to two extractions with methanol (70%, *v/v*) supported by ultrasound (20 min, 30 °C, 50 Hz) (Sonic 10, Polsonic, Poland). The collected supernatants were combined, evaporated (R-215 Rotavapor System, Buchi, Switzerland) and applied to a LiChroprep RP-18 bed (40-63 µm). Polyphenolic compounds were washed with methanol, concentrated using an evaporator and freeze-dried.

#### **Determination of Polyphenols Profile by UPLC-ESI-TQD-MS/MS**

Hawthorn seed extracts were dissolved in 50% methanol. Polyphenolic compounds were identified using an ultra-performance liquid chromatograph (UPLC) equipped with a photodiode detector (PDA), a TQD mass spectrometer with an electrospray ionization (EPI) source (Waters, Milford, MA, USA). The separation of polyphenols was carried out on a UPLC BEH C18 column (1.7 µm, 100 x 2.1 mm Waters, Warsaw, Poland) at a temperature of 50 ^o^C with an injection volume of 5 µL and a flow of 0.35 mL/min. The mobile phase consisted of solvent A (water) and solvent B (40% acetonitrile). The gradient program was set as follows: 0 min 5% B, from 0 to 8 min linear to 100% B, and from 8 to 9.5 min for washing and returning to the initial conditions. The optimized TQD parameters were as follows: nitrogen flow rate 100 L/h, cone voltage 30 V, capillary voltage 3500 V, source and dissolution temperature 120 °C and 350 °C, respectively. The obtained data were processed using MassLynx v.4.1 Waters, Milford, MA, USA). Compounds were identified based on retention time, molecular weight, UV-VIS spectrum, MS/MS ions and literature data [1, 3–8]. For quantitative analysis the PDA chromatograms have been used. The individual peaks have been integrated at 270 nm for condensed tanins, 320 nm and 350 nm for phenolic acid and flavonols respectively. Quantification was performed using calibration curves made for the following standards: keampferol-3-*O*-glucose, quercetin-3-*O*-rutinoside, *p*-coumaric acid, 3-*O*-caffeoylquinic acid, 5-*O*-caffeoylquinic acid, (+)-catechin in concentrations in the range from 0.05 to 0.5 mg/mL (R2 ≤ 0.9998). All condensed tannins have been quantified according to (+)Catechin as a standard. Results are expressed in mg/g d.w.

**Table S1:** Calibration curve parameters of the method developed for each standard.

| **No** | **Compound** | **Linearity range**  **[µg/mL]** | **Regresion equation** | **R2** |
| --- | --- | --- | --- | --- |
| 1 | 3-*O*-caffeoylquinic acid | 50-500 | y = 3.84x10^-5^ + 0.27 | 0.998 |
| 2 | 5-*O*-caffeoylquinic acid | 50-500 | y = 3.06x10^-5^ + 0.32 | 0.999 |
| 3 | *p*-coumaric acid | 50-500 | y = 1.95x10^-5^ + 0.67 | 0.999 |
| 4 | (+)Catechin | 50-500 | y = 5.16x10^-5^ – 2.40 | 0.999 |
| 5 | Quercetin 3-*O*-rutinoside | 50-500 | y = 1.28x10-^5^ – 0.34 | 0.999 |
| 6 | Kaempferol 3-*O*-glucoside | 50-500 | y = 1.19x10^-5^ – 0.27 | 0.999 |

***Determination of Antioxidant Activity***

To determine the antioxidant properties, the powders were dissolved in 50% methanol (5 mg/ mL).

The antioxidant activity was assessed by measuring ABTS^•^*^+^* radical scavenging activity [9], copper ion reduction by CUPRAC method [10], metal chelating capacity (ChP) [11], **superoxide**(O_2_^•-^) and hydroxyl (OH^•^) **radicals scavenging activity** [12]**.** In the assessment metal chelating capacity, **superoxide**(O_2_^•-^) and hydroxyl (OH^•^) **radicals scavenging activity** the concentrations of extracts in the range were used 10-500 μg/mL seed extracts. The results were expressed as Trolox Equivalent (mmol TE/g d.w.) and IC_50_ (μg/mL).

***In vitro anti-obesity, -diabetic and -trypsin activity***

Before testing, 2 mg of hawthorn seed extracts were dissolved in 100 µL of 50% methanol to ensure complete dissolution of the active compounds. Then, 900 µL of water was added to achieve a final concentration of 2 mg/mL. The inhibitory properties of the powdered extracts were measured using extracts at a working concentration prepared by diluting the initial solution (2 mg/mL) 20 to 50 times. This range was chosen to ensure a reduction in enzymatic activities within the range of 20% to 80% of the control.

The inhibitory activities of lipase were measured using 4-nitrophenyl acetate as a substrate [13]. The reaction mixture contained 220 μL of 100 mmol/L TRIS-HCL buffer pH 7.6, 10 μL of the enzyme (10 μg/mL, pancreatin from porcine pancreas, 4 × UPS, P1750 Sigma-Aldrich, Poland) and 20 μL of the substrate (10 mmol/L). The change of absorbance (3 min) was measured at 410 nm. For the inhibition studies, before adding the substrate the enzyme was incubated for 10 min with 10 μL of the studied extract. One unit of lipase activity releases 1 μmol of 4-nitrophenyl per minute at pH 7.6 at 25 °C.

The inhibitory activities of α-amylase were measured using Red-starch as a substrate [14]. The reaction mixture contained 120 μL of 50 mmol/L phosphate buffer pH 6.6 containing 6 mmol/L NaCl, 10 μL of the enzyme (10 ug/mL, α-amylase from porcine pancreas, 50 U/mg, A3176 Sigma-Aldrich, Poland) and 20 μL of the substrate (2%). The reaction mixture was incubated for 20 min at 40 °C and stopped by adding 150 μL of pure ethanol. After centrifugation (15 min, 25 °C, 5000 g) 150 μL was transferred into new plates and absorbance was measured at 510 nm using. For the inhibition studies, before adding the substrate the enzyme was incubated for 10 min with 20 μL of the studied extract. One unit of activity will liberate 1.0 μmol of maltose in 1 min at reaction conditions.

The inhibitory activities of trypsin were measured using N-α-Benzoyl-L-arginine 4-nitroanilide hydrochloride as a substrate [15]. The reaction mixture contained 220 μL of 100 mmol/L TRIS-HCL buffer pH 7.6, 10 μL of the enzyme (10 μg/mL, α-trypsin from bovine pancreas, 800 U/mg, T7409 Sigma-Aldrich, Poland) and 20 μL of the substrate (20 mmol/L). The change of absorbance (3 min) was measured at 410 nm. For the inhibition studies, before adding the substrate the enzyme was incubated for 10 min with 10 μL of the studied extract. One unit releases of 1 μmol of 4-nitroanilide per minute at pH 7.6 at 37 °C.

All inhibitory assay were read using in a BioTek Epoch microplate reader. The activity is expressed in IU/g d.w., where IU is defined as an amount of inhibitor decreasing an 1 U of enzyme activity.

***In vitro anti-inflammatory activity***

The inhibitory activities of lipoxygenase (LOX) was measured using linoleic acid as a substrate [16]. The reaction mixture contained 250 μL of 1/15 M sodium-phosphate buffer, 10 μl of the enzyme (10 μg/mL, L7395 Sigma-Aldrich, Poland) and 40 μL of the substrate (5 mmol/L). The change of absorbance (3 min) was measured at 252 nm. For the inhibition studies, before adding the substrate the enzyme was incubated for 10 min with 10 μL of the studied extract. One unit (U) will cause an oxidation of 1 μmol of linoleic acid per minute at pH 7.5 at 30 °C.

The inhibitory activities of cyclooxygenase-2 (COX-2) were measured using the COX Colorimetric Inhibitor Screening Assay Kit (701050, Cayman Chemicals). One unit of enzyme activity causes the oxidation of 1 nmol of N,N,N',N'-tetramethyl-*p*-phenylenediamine (TMPD) per min at pH 7.5 at 25 °C.

The inhibitory activities of xanthine oxidase (XO) was measured using xanthine as a substrate [16]. The reaction mixture contained 120 μL of 1/15 M sodium-phosphate buffer, 20 μL of the enzyme xanthine oxidase (10 μL/mL, X1875 Sigma-Aldrich, Poland) and 20 μL of the substrate (0.015 mmol). The change of absorbance (3 min) was measured at 234 nm. For the inhibition studies, before adding the substrate the enzyme was incubated for 10 min with 20 μL of the studied extract. One unit of activity converts 1.0 μmol of xanthine to uric acid per min at pH 7.5 at 25 °C.

All inhibitory assays were read using a BioTek Epoch microplate reader. The activity is expressed in IU/g d.w., where IU is defined as an amount of inhibitor decreasing a 1 U of enzyme activity.

***Cell Culture***

Eleven cell lines were selected for the study: breast cancer cell line (Mcf-7), glioblastoma (U87mg), astrocytoma (U251mg), gastric cancer (AGS), four colorectal cancer cell lines (Ht-29, Caco-2, Ls180, Dld-1), melanoma (Sk-mel-28), leukemic T-cell lymphoblast (Jurkat), and one line of healthy colonic epithelial cells (CCD841CoN). Cell lines were cultured in DMEM, McCoy's and RPMI media supplemented with fetal bovine serum (10%) and the antibiotics penicillin/streptomycin (1%). Cell growth was ensured by maintaining the following conditions: 5% CO_2_ atmosphere, 37 °C, 95% humidity (CB170, Binder, Tuttlinen, Germany).

**Cell Viability Assay**

Cell viability was assessed by a standard method using the MTS assay (Promega). All cell lines were seeded in 96-well plates (8 x 10^4^ cells/well) and incubated at 37 ^o^C for 24 h. The cells were then exposed to 24-hour exposure to seed extracts (10-750 μg/mL). After this time, the extracts were replaced with fresh medium, CellTiter 96 ^®^ AQ _ueous_ One Solution Cell Proliferation Assay reagent was added and the color change was measured at a wavelength of 490 nm using a microplate reader (SmartReader 96 Microplate Absorbance Reader, Accuris Instruments, Edison, USA). The results were expressed as the IC_50_ (μg/mL).

**Statistical Analysis**

Statistical analysis, including one-way Duncan's test (*p* <0.05), Pearson's correlation (*p* <0.05), principal components analysis (PCA) was performed in Statistica 13.3 software (StatSoft, Krakow, Poland).

**Table S2**. The content of phenolic compounds (mg/g) identified in the seeds of six hawthorn species.

| **Peak No** | **Compound** | **Species of hawthorn** | | | | | |
| --- | --- | --- | --- | --- | --- | --- | --- |
|  |  | *C. monogyna* | *C. rhipidophylla* | *C. x subsphaericea* | *C. laevigata x rhipidophylla x monogyna* | *C. macrocarpa* | *C. laevigata* |
| 1 | 5*-O*-caffeoylquinic acid | 1.3 ± 0.0^b^ | 1.3 ± 0.0^b^ | 1.7 ± 0.0^c^ | 0.7 ± 0.0^a^ | 2.1 ± 0.0^d^ | 0.7 ± 0.0^a^ |
| 3 | Coumarylquinic acid | 1.4 ± 0.0^c^ | 0.3 ± 0.0^a^ | 1.6 ± 0.0^d^ | 1.4 ± 0.0^c^ | 2.2 ± 0.0^e^ | 0.7 ± 0.0^b^ |
| 4 | 3-*O*-caffeoylquinic acid | 1.2 ± 0.0^c^ | 0.4 ± 0.0^a^ | 1.6 ± 0.1^d^ | 1.0 ± 0.0^b^ | 3.5 ± 0.1^f^ | 3.1 ± 0.0^e^ |
|  | ***Sum phenolic acid*** | 3.8 ± 0.1^c^ | 2.1 ± 0.1^a^ | 5.0 ± 0.1^e^ | 3.2 ± 0.1^b^ | 7.7 ± 0.2^f^ | 4.6 ± 0.1^d^ |
| 2 | Procyanidin dimer type-A | 0.8 ± 0.0^c^ | 0.3 ± 0.0^a^ | 0.4 ± 0.0^b^ | 0.4 ± 0.0^b^ | 1.4 ± 0.0^d^ | 2.1 ± 0.0^e^ |
| 5 | Procyanidin trimer | 1.6 ± 0.1^a^ | 1.8 ± 0.1^c^ | 1.7 ± 0.0^b^ | 2.8 ± 0.1^e^ | 2.4 ± 0.0^d^ | 1.6 ± 0.1^a^ |
| 6 | Procyanidin dimer type-B | 7.5 ± 0.0^c^ | 7.2 ± 0.2^b^ | 9.0 ± 0.0^d^ | 9.3 ± 0.0^e^ | 10.9 ± 0.1^f^ | 6.3 ± 0.1^a^ |
| 7 | Procyanidin dimer type-B | 1.3 ± 0.1^a^ | 1.6 ± 0.0^b^ | 2.3 ± 0.1^c^ | 2.5 ± 0.1^e^ | 2.4 ± 0.1^d^ | 1.4 ± 0.0^a^ |
| 8 | (+)Catechin | 9.5 ± 0.1^a^ | 11.3 ± 0.1^d^ | 9.7 ± 0.1^b^ | 11.0 ± 0.1^c^ | 11.9 ± 0.1^e^ | 11.3 ± 0.0^d^ |
| 9 | Proanthocyanidin dimer | 1.5 ± 0.1^cd^ | 1.5 ± 0.0^d^ | 0.9 ± 0.1^a^ | 1.0 ± 0.0^b^ | 1.4 ± 0.1^c^ | 1.8 ± 0.0^e^ |
| 10 | Procyanidin trimer | 6.6 ± 0.1^b^ | 7.4 ± 0.0^c^ | 8.7 ± 0.3^d^ | 9.0 ± 0.1^d^ | 10.2 ± 0.2^e^ | 6.0 ± 0.0^a^ |
| 11 | Procyanidin dimer type-B | 3.4 ± 0.0^b^ | 4.3 ± 0.1^c^ | 5.8 ± 0.0^d^ | 5.9 ± 0.1^e^ | 6.6 ± 0.0^f^ | 2.9 ± 0.1^a^ |
| 12 | Proanthocyanidin pentamer | 1.9 ± 0.1^b^ | 2.3 ± 0.1^c^ | 3.4 ± 0.1^d^ | 3.9 ± 0.2^e^ | 3.3 ± 0.1^d^ | 1.6 ± 0.1^a^ |
| 13 | Procyanidin trimer | 1.8 ± 0.1^a^ | 2.3 ± 0.1^b^ | 4.0 ± 0.2^cd^ | 4.1 ± 0.2^d^ | 3.9 ± 0.2^c^ | 1.9 ± 0.0^a^ |
| 14 | Proanthocyanidin pentamer | 0.4 ± 0.0^b^ | 0.3 ± 0.0^a^ | 0.3 ± 0.0^a^ | 0.6 ± 0.0^d^ | 0.5 ± 0.0^c^ | 0.4 ± 0.0^b^ |
| 15 | Procyanidin trimer | 0.5 ± 0.0^b^ | 0.3 ± 0.0^a^ | 0.3 ± 0.0^a^ | 0.5 ± 0.0^b^ | 0.7 ± 0.0^c^ | 1.1 ± 0.0^d^ |
| 16 | Procyanidin dimer type-B | 0.8 ± 0.0^c^ | 0.6 ± 0.0^b^ | 1.5 ± 0.0^d^ | 3.0 ± 0.0^e^ | 1.5 ± 0.0^d^ | 0.4 ± 0.0^a^ |
| 17 | Procyanthocyanidin trimer | 1.4 ± 0.0^e^ | 1.1 ± 0.1^c^ | 0.7 ± 0.0^a^ | 1.3 ± 0.1^d^ | 1.0 ± 0.0^b^ | 1.2 ± 0.1^c^ |
| 18 | (Epi)afzelechin-di-hexoside | 0.5 ± 0.0^c^ | 1.1 ± 0.0^f^ | 0.7 ± 0.0^d^ | 0.3 ± 0.0^b^ | 1.0 ± 0.0^e^ | 0.1 ± 0.0^a^ |
| 19 | Unspecified (Epi)afzelechin derivative | 0.9 ± 0.0^c^ | 1.3 ± 0.0^d^ | 0.9 ± 0.0^c^ | 0.4 ± 0.0^b^ | 1.3 ± 0.0^d^ | 0.2 ± 0.0^a^ |
|  | ***Sum flavan-3-ols*** | 40.4 ± 0.7^a^ | 45.1 ± 0.9^b^ | 50.4 ± 1.0^c^ | 56.2 ± 0.9^d^ | 60.3 ± 1.0^e^ | 40.4 ± 0.6^a^ |
| 20 | Kaempferol-*O*-galloyl-pentoside isomer I | 0.8 ± 0.0^b^ | 1.1 ± 0.0^de^ | 1.0 ± 0.0^c^ | 1.1 ± 0.0^cd^ | 1.1 ± 0.0^e^ | 0.6 ± 0.0^a^ |
| 21 | Kaempferol-*O*-galloyl-pentoside isomer II | 1.0 ± 0.0^b^ | 1.4 ± 0.0^e^ | 1.3 ± 0.0^d^ | 1.2 ± 0.0^cd^ | 1.2 ± 0.0^c^ | 0.6 ± 0.0^a^ |
| 22 | Quercetin *O*-acetyl-hexoside | 0.7 ± 0.0^b^ | 0.9 ± 0.0^c^ | 0.9 ± 0.0^c^ | 1.1 ± 0.0^d^ | 0.7 ± 0.0^b^ | 0.5 ± 0.0^a^ |
| 23 | Unspecified | 0.2 ± 0.0^b^ | 0.3 ± 0.0^d^ | 0.4 ± 0.0^e^ | 0.4 ± 0.0^f^ | 0.3 ± 0.0^c^ | 0.2 ± 0.0^a^ |
|  | ***Sum flavonols*** | 2.8 ± 0.1^b^ | 3.6 ± 0.1^d^ | 3.6 ± 0.1^d^ | 3.8 ± 0.1^e^ | 3.3 ± 0.1^c^ | 1.8 ± 0.0^a^ |
|  | **TOTAL [mg/g]** | **47.1 ± 0.9^a^** | **50.8 ± 1.0^b^** | **58.9 ± 1.2^c^** | **63.1 ± 1.1^d^** | **71.3 ± 1.3^e^** | **46.8 ± 0.7^a^** |

Results are expressed as mean and SD. Significant differences between species were assessed by Duncan's test (*p* <0.05).

**References**

1. Żurek, N., Karatsai, O., Rędowicz, M.J., Kapusta, I.T.: Polyphenolic Compounds of Crataegus Berry, Leaf, and Flower Extracts Affect Viability and Invasive Potential of Human Glioblastoma Cells. Molecules. 26, 2656 (2021). https://doi.org/10.3390/molecules26092656.

2. Żurek, N., Kapusta, I., Cebulak, T.: Impact of extraction conditions on antioxidant potential of extracts of flowers, leaves and fruits of Hawthorn (Crataegus × macrocarpa l.). Food, Sci. Technol. Qual. 27, 130–141 (2020). https://doi.org/10.15193/zntj/2020/123/340.

3. Badalica-Petrescu, M., Dragan, S., Ranga, F., Fetea, F., Socaciu, C.: Comparative HPLC-DAD-ESI(+)MS Fingerprint and Quantification of Phenolic and Flavonoid Composition of Aqueous Leaf Extracts of *Cornus mas* and *Crataegus monogyna*, in Relation to Their Cardiotonic Potential. Not Bot Hort Agrobot Cluj. 42, 9–18 (2014). https://doi.org/10.15835/nbha4219270.

4. Elsadig Karar, M.G., Kuhnert, N.: UPLC-ESI-Q-TOF-MS/MS Characterization of Phenolics from Crataegus monogyna and Crataegus laevigata (Hawthorn) Leaves, Fruits and their Herbal Derived Drops (Crataegutt Tropfen). J Chem Biol Ther. 01, (2016). https://doi.org/10.4172/2572-0406.1000102.

5. González-Jiménez, F.E., Salazar-Montoya, J.A., Calva-Calva, G., Ramos-Ramírez, E.G.: Phytochemical Characterization, *In Vitro* Antioxidant Activity, and Quantitative Analysis by Micellar Electrokinetic Chromatography of Hawthorn ( *Crataegus pubescens* ) Fruit. Journal of Food Quality. 2018, 1–11 (2018). https://doi.org/10.1155/2018/2154893.

6. Liu, P., Kallio, H., Lü, D., Zhou, C., Yang, B.: Quantitative analysis of phenolic compounds in Chinese hawthorn (Crataegus spp.) fruits by high performance liquid chromatography–electrospray ionisation mass spectrometry. Food Chemistry. 127, 1370–1377 (2011). https://doi.org/10.1016/j.foodchem.2011.01.103.

7. Pawłowska, A.M., Żurek, N., Kapusta, I., De Leo, M., Braca, A.: Antioxidant and Antiproliferative Activities of Phenolic Extracts of Eriobotrya japonica (Thunb.) Lindl. Fruits and Leaves. Plants. 12, 3221 (2023). https://doi.org/10.3390/plants12183221.

8. Żurek, N., Kapsuta, I., Cebulak, T.: Content of Polyphenolic Compounds and Biological Activity of Berries, Leaves and Flowers of *Crataegus* L. Acta Universitatis Cibiniensis. Series E: Food Technology. 27, 35–52 (2023). https://doi.org/10.2478/aucft-2023-0004.

9. Re, R., Pellegrini, N., Proteggente, A., Pannala, A., Yang, M., Rice-Evans, C.: Antioxidant activity applying an improved ABTS radical cation decolorization assay. Free Radic Biol Med. 26, 1231–1237 (1999). https://doi.org/10.1016/S0891-5849(98)00315-3.

10. Apak, R., Güçlü, K., Özyürek, M., Esin Karademir, S., Erçağ, E.: The cupric ion reducing antioxidant capacity and polyphenolic content of some herbal teas. Int J Food Sci Nutr. 57, 292–304 (2006). https://doi.org/10.1080/09637480600798132.

11. Żurek, N., Pycia, K., Pawłowska, A., Kapusta, I.T.: Phytochemical Screening and Bioactive Properties of Juglans regia L. Pollen. Antioxidants. 11, 2046 (2022). https://doi.org/10.3390/antiox11102046.

12. Żurek, N., Pawłowska, A., Kapusta, I.: Obtaining preparations with increased content of bioactive compounds from eight types of berries. JBR. 1–17 (2023). https://doi.org/10.3233/JBR-230020.

13. Swieca, M., Gawlik-Dziki, U., Jakubczyk, A., Bochnak, J., Sikora, M., Suliburska, J.: Nutritional quality of fresh and stored legumes sprouts – Effect of Lactobacillus plantarum 299v enrichment. Food Chemistry. 288, 325–332 (2019). https://doi.org/10.1016/j.foodchem.2019.02.135.

14. Lachowicz, S., Kapusta, I., Świeca, M., Stinco, C.M., Meléndez-Martínez, A.J., Bieniek, A.: In Vitro Biological Activities of Fruits and Leaves of Elaeagnus multiflora Thunb. and Their Isoprenoids and Polyphenolics Profile. Antioxidants. 9, 436 (2020). https://doi.org/10.3390/antiox9050436.

15. Świeca, M., Baraniak, B., Gawlik-Dziki, U.: In vitro digestibility and starch content, predicted glycemic index and potential in vitro antidiabetic effect of lentil sprouts obtained by different germination techniques. Food Chemistry. 138, 1414–1420 (2013). https://doi.org/10.1016/j.foodchem.2012.09.122.

16. Lachowicz, S., Świeca, M., Pejcz, E.: Biological activity, phytochemical parameters, and potential bioaccessibility of wheat bread enriched with powder and microcapsules made from Saskatoon berry. Food Chemistry. 338, 128026 (2021). https://doi.org/10.1016/j.foodchem.2020.128026.
